# Hip and Knee Bracing: Categorization, Treatment Algorithm, and Systematic Review

**DOI:** 10.5435/JAAOSGlobal-D-20-00181

**Published:** 2021-06-07

**Authors:** Bernard P. Kemker, Roma Kankaria, Nirav Patel, Gregory Golladay

**Affiliations:** From the Virginia Commonwealth University Medical Center, Richmond, VA.

## Abstract

Hip and knee braces or orthoses are often used to provide support after surgery and to prevent or reduce the severity of injuries. The braces are used for stabilization, immobilization, mechanical correction, and rehabilitation. Hip braces consist of stabilization and unloader variations, whereas knee braces are composed of knee sleeves and patellofemoral, prophylactic, unloader, and functional braces. Indications vary widely and depend on the type of brace. Hip braces can treat osteoarthritis to instability after total hip arthroplasty. Knee brace indications range from mild arthralgias to instability and osteoarthritis. Although braces are routinely used clinically, high-level evidence is sparse for their use. With this review, the different types and uses of hip and knee braces have been defined, and their indications exemplified in hopes of spurring future research.

Hip and knee braces do not have high-level evidence. Yet, barely a clinic goes by that must orthopaedic surgeons do not use or offer consultation on bracing treatment. Furthermore, a review of the past Orthopaedic In-Training Online Examinations since 2012 only revealed 12 questions about adult hip and knee braces. Do not worry; sole bracing treatment was the correct answer only once, when used in combination of suture fixation of the medial collateral ligament (MCL) during total knee arthroplasty (TKA). Bracing treatment follows many similarities with multiple other nonsurgical treatments with concerns of cost, efficacy, and patient compliance dictating treatment protocols. However, even a basic description or recommendation of their general uses is not present in orthopaedic literature since the turn of the 21st century.

Hip braces are less commonly used than knee braces. However, hip braces provide a powerful tool in the face of dislocation after total hip arthroplasty (THA).^1^ In addition, surgeons use hip braces for postoperative protection after hip arthroscopy in protection of the labral repair or osteoplasties. The more commonly used knee brace helps in knee stabilization, immobilization, proprioception, and realignment.^1^ Knee braces fall into six categories, (1) prophylactic knee braces, (2) functional knee braces, (3) unloader braces, (4) patellofemoral braces, (5) rehabilitative braces, and (6) knee sleeves (Table 1). Interestingly, prophylactic braces, functional braces, and rehabilitative braces may be the same off-the-shelf (OTS) brace, but each treats a different diagnosis whether used postoperatively or for a high-level athlete. This can cause much confusion within the medical field. Thus, we offer a review of hip and knee braces with their indications and a treatment algorithm (Figure 1) for easier clinical use.

**Table 1:** Indications and Examples of Each Type of Knee Brace

Type	Indication
Prophylactic	Valgus knee stress; rotational stress; MCL injury; high risk for MCL injury
Functional	Unstable, injured knees; ACL injury; MCL instability; PCL instability
Unloader	Knee osteoarthritis
Patellofemoral	Patellofemoral pain
Rehabilitative	Injured knee; surgically repaired knee
Sleeve	Acute knee injuries; flares of knee osteoarthritis; knee pain or dysfunction

ACL = anterior cruciate ligament, MCL = medial collateral ligament, PCL = posterior cruciate ligament

**Figure 1:**
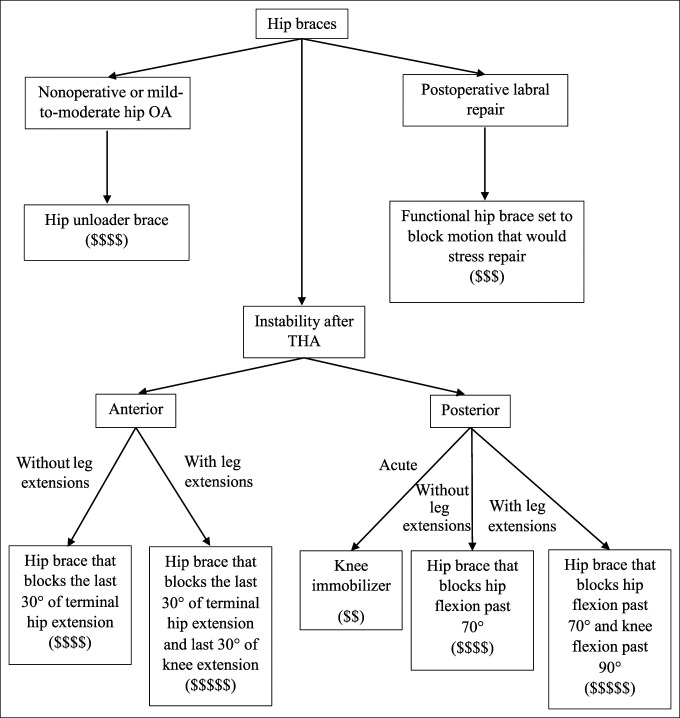
Flowchart showing the hip brace indications and treatment schematic.

## Hip Braces

One of the primary functions of a hip brace is to hold the hip in a reduced position after dislocation, whether it be implants after a THA or the native joint.^1^ With the emergence of hip arthroscopy, hip braces are also used for postoperative range of motion restriction after labral repairs or osteoplasties about the hip; however, postoperative rehabilitation protocols are infrequently reported, and most certainly not rigorously studied.^2^ Thus, the use of hip braces for postoperative rehabilitation remains surgeon-specific. Indications and examples of hip braces are listed in Table 2.

**Table 2:** Indications and Examples of Each Type of Hip Brace

Type	Indication
Stabilizer	Hip dislocation
Knee immobilizer	Hip dislocation
Unloader	Hip osteoarthritis

**Figure 2:**
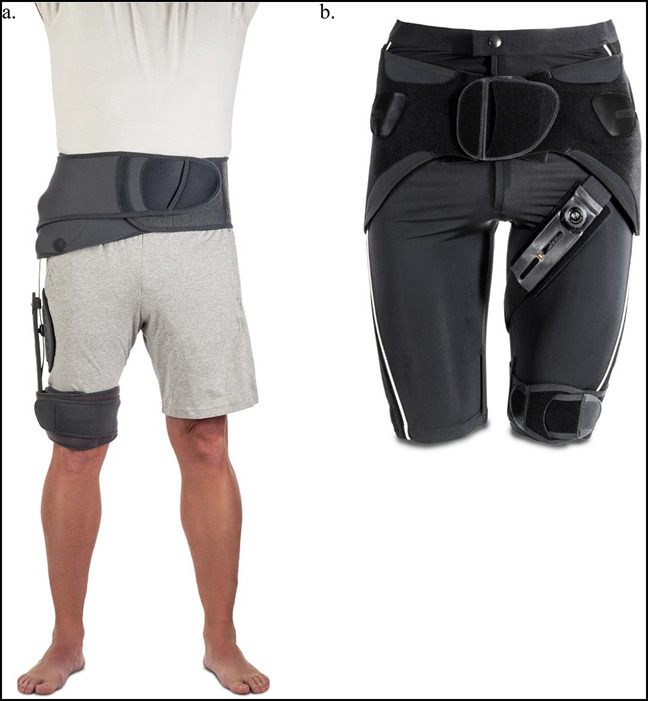
Photographs showing the hip braces. The stabilizing hip brace (**A**) (Ӧssur Rebound Hip Brace) and hip unloader brace (**B**) (Ӧssur Unloader Hip Brace) are depicted. The hip-stabilizing brace is secured to the trunk and distal thigh; the “stays” can limit hip abduction, adduction, flexion, and extension. This brace is commonly used to restrict motion after total hip arthroscopy (THA) or for hip instability after THA. The hip unloader brace transfers patient's body weight away from the diseased cartilage in their osteoarthritic hip (permission to use these photographs was received from Ӧssur).

**Figure 3:**
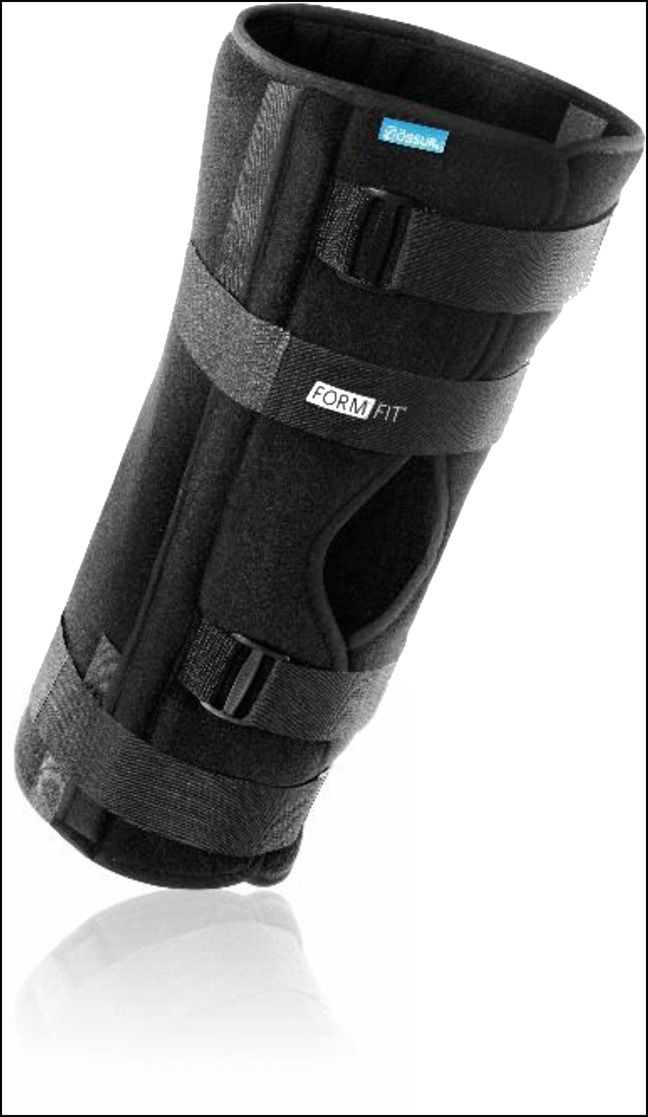
Photograph showing the knee immobilizer brace (Ӧssur Formfit). The medial and lateral “stays” are secured to the patient's lower limb with Velcro straps. This prevents any varus-valgus stress and prevents knee flexion while holding the knee in full extension to slight hyperextension (permission to use this photograph was received from Ӧssur, Reykjavík, Iceland).

Hip bracing use for instability after THA is controversial. Although the use of a hip brace for continuous treatment for hip instability is poorly tolerated and is not definitive solution for recurrent hip dislocations, hip braces are used in case of acute hip dislocations.^3,4^ As depicted in Figure 2A, the stabilizing hip brace can be positioned above the knee and limit hip flexion. Decreasing hip flexion prevents hip impingement that can cause a posterior dislocation. For the acute hip dislocation after a posterior THA, a simple knee immobilizer can be used (Figure 3). The immobilizer blocks knee flexion and hip flexion by 50% by tightening the hamstrings. With the hamstrings taut, hip flexion is decreased, which prevents posterior dislocation. Although this is cumbersome to wear and often slide down, the knee immobilizer is better than the use of the traditional hip brace. Although compliance is not perfect, it is better than the use of the traditional hip brace.^5^ A knee immobilizer and traditional hip brace can be combined, or a custom hip brace can be made to limit hip flexion, knee flexion, and hip internal rotation.

Importantly, note that although a knee immobilizer is a reasonable initial treatment in the posterior dislocation of the THA, a knee immobilizer can exacerbate anterior hip instability. A stabilizing hip brace is required to block hip the terminal 30^o^ of hip extension. In addition, if extra support is required, the brace can be extended to below the knee and block the terminal 30° of knee extension, keeping the quadriceps and anterior structures taut and preventing posterior impingement, which can cause an anterior dislocation.^6^ The same can be done for the posterior THA dislocation, but hip flexion and knee flexion are blocked,^1^ which is depicted in Figure 1. Despite compliance being an issue, some patients are not able to undergo revision surgery, thus justifying the use of a hip brace.

Unloader hip braces facilitate load dispersion for patients with hip osteoarthritis (Figure 2B). The braces improve patient mobility while offering more stability to the hips. Clinical practice guidelines published by the *American Academy of Orthopaedic Surgeons* (AAOS) do not include a recommendation for or against the use of hip braces in the treatment of hip osteoarthritis.^7^ For patients who are not surgical candidates, a hip unloader brace can be used to relieve pain; however, compliance remains low because they are uncomfortable to wear. A treatment schematic for all hip braces is listed in Figure 1.

## Knee Braces

### Prophylactic

The purpose of prophylactic knee braces is to prevent or reduce the severity of ligamentous knee injuries. Indications include MCL protection during a valgus knee stress, protection of reinjury after a primary MCL injury, and protection for patients who are at high risk for MCL injury. Although prophylactic knee braces are commonly used in contact sports, such as football, prophylactic braces have not consistently reduced MCL injuries and lack evidence for routine use in uninjured knees.^8^ Although these braces may be beneficial in protecting against varus-valgus knee stresses in contact sports, athletes who play with frequent rotational moments on the knee may be safer without wearing the brace.^9^

**Figure 4:**
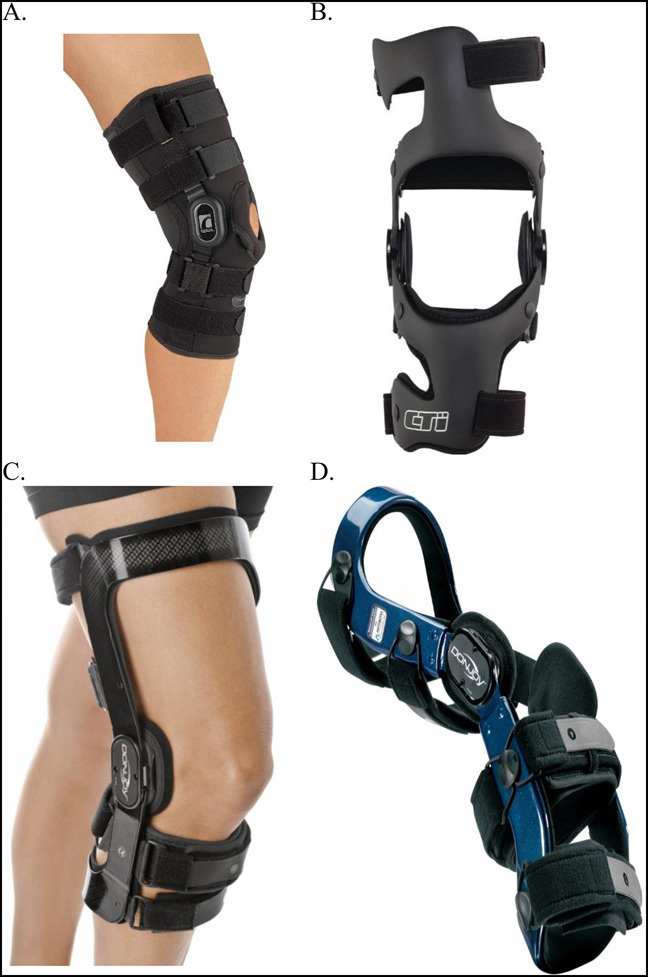
Photographs showing the prophylactic knee braces. The Ӧssur Rebound (**A**), the Ӧssur CTi (**B**), the DonJoy Fullforce (**C**), and the DonJoy Defiance (**D**) are demonstrated. These braces increase in complexity from **A** to **B**, with both preventing varus-valgus motion by medial and lateral stays (metal in **A** and carbon fiber in **B**, **C**, and **D**) and containing a central hinge to limit knee range of motion. Knee braces similar **A** and **C** are off-the-shelf braces and routinely used postoperatively to limit knee range of motion. Knee braces similar to **B** and **D** can be additionally customized to a wide variety of knee uses such as anterior cruciate ligament, posterior cruciate ligament, medial collateral ligament, and LCL prophylaxis or treatment after injury. An additional benefit to **C** and **D** is that these braces can also be modified into a valgus unloader brace (permission to use these photographs were received from Ӧssur, Reykjavík, Iceland, and DJO Lewisville TX).

Two different mechanisms of action exist for prophylactic braces, relating to the two stabilizing structures involved. The mechanical support for knee braces is provided by unilateral and bilateral supports or stays. A unilateral support brace has a medial or lateral upright stay. The stay can be single-axis, dual-axis, or polycentric. Bilateral support braces have a stay on the medial and lateral side, which can be bilateral or polycentric. Examples of prophylactic braces are the Ӧssur Rebound (Össur), the Össur CTi (Össur), the DonJoy Fullforce (DJO), and the DonJoy Defiance (DJO), shown in Figure 4.

The braces can limit excessive, post-reconstruction tibial rotation from pivoting during sporting activities.^10^ Some studies have shown that the braces may decrease the risk of noncontact knee injuries in sporting activities.^11^ In addition, prophylactic knee braces may stabilize the knee joint in the landing phase of athletes' dynamic movements by increasing the stiffness of the hamstrings.^12^ However, other studies have shown no difference in the number of knee injuries in athletes who wore the prophylactic brace compared with those who did not.^13^ Because of the conflicting evidence on efficacy, the routine use of prophylactic knee braces is not recommended.

### Functional

Functional knee braces provide stability for unstable, injured knees, especially after anterior cruciate ligament (ACL) injury. In addition to supporting the knee after ACL reconstructive surgery, the brace can be used to address MCL or posterior cruciate ligament (PCL) instability. A functional knee brace can facilitate rehabilitation after MCL and PCL injury. It can also be used to protect MCL and PCL repair/reconstruction postoperatively to aid healing. For arthroplasty surgeons, the use of a brace postoperatively is usually not indicated except when the MCL is incompetent or injured during TKA. Although increasing constraint is always an option, maintaining a lower level of constraint with MCL repair combined with the use of a functional brace is preferred. Many functional braces still allow for full flexion and extension and do not limit postoperative physical rehabilitation.

The mechanism of action for functional knee braces, which can be a custom fit or presized (OTS) fit, relies on double-hinged bars with range of motion stops and straps that have fitted cuffs or shells. The double-hinged bars provide support in the coronal plane and prevent excessive varus-valgus stress. The brace is designed to reduce the anterior and posterior tibial translation to restore normal motion after knee injury, with range of motion stops as needed. Additional reductions in knee strain can be achieved during motions such as posterior shear loading, tibial rotation, and tibial translation.^14,15^

**Figure 5:**
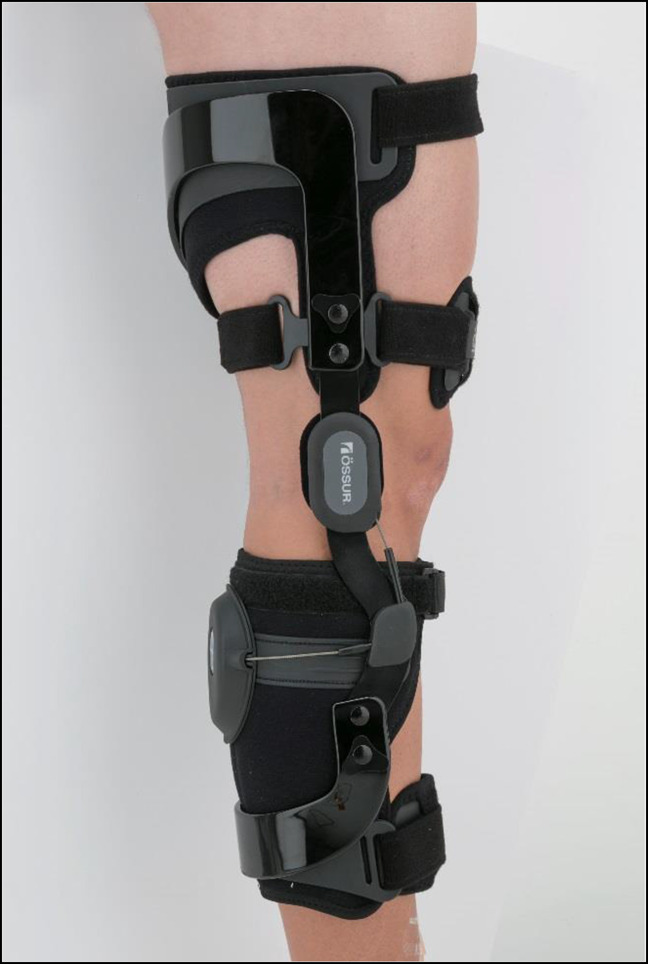
Photograph showing the functional posterior cruciate ligament brace (Ӧssur Rebound PCL Knee Brace). The brace is similar to those in Figure 3 with the inclusion of a posterior tibial restraint that prevents posterior subluxation of the tibia under the femur. This, in turn, prevents or decreases the load transmitted to the posterior cruciate ligament. Indications include unstable knees, anterior cruciate ligament injury, medial collateral ligament instability, and posterior cruciate ligament instability (permission to use this photograph was received from Ӧssur, Reykjavík, Iceland).

**Figure 6:**
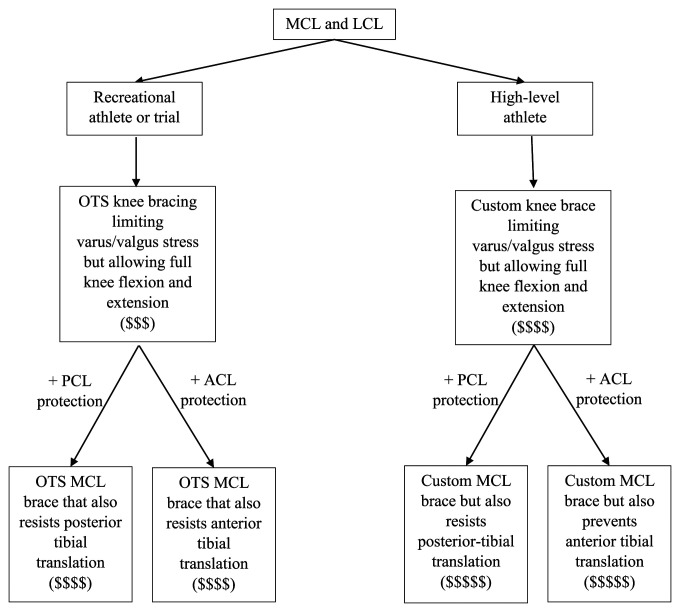
Flowchart showing the prophylactic knee brace treatment schematic.

**Figure 7:**
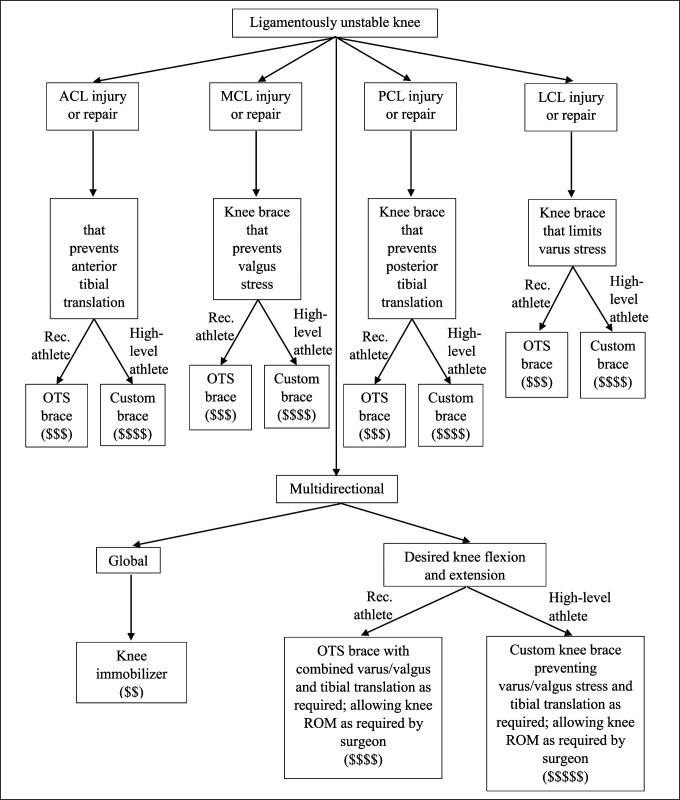
Flowchart showing the functional brace schematic.

Another example of functional knee braces is the Rebound PCL Knee Braces (Össur) depicted in Figure 5. Functional PCL knee braces allow the ligament to effectively heal with less attenuation.^16^ Dynamic functional braces apply increasing posteriorly directed force to the tibia, but additional studies are needed to determine whether they result in long-term improvements to mechanical stability after PCL injury^17^ (Figure 6). In addition, more modern functional braces can be manufactured patient-specifically (custom-made) for cruciate ligament protection, such as the Ӧssur CTi (Össur) and the DonJoy Defiance (DJO), shown in Figure 4. Figure 7 illustrates a treatment schematic for functional and postoperative braces.

### Unloader

Clinical practice guidelines published by the AAOS for knee osteoarthritis include moderate-to-strong recommendations for bracing treatment in early pain management. However, the data are not conclusive, and the guidelines are unable to recommend the use of medial compartment unloader braces.^18^ Despite this, unloader knee braces have been shown to reduce knee pain for patients with knee osteoarthritis who need to be treated nonoperatively because of patient's choice or other reasons such as medical comorbidities.^19^ Therefore, valuable treatment options still remain.

**Figure 8:**
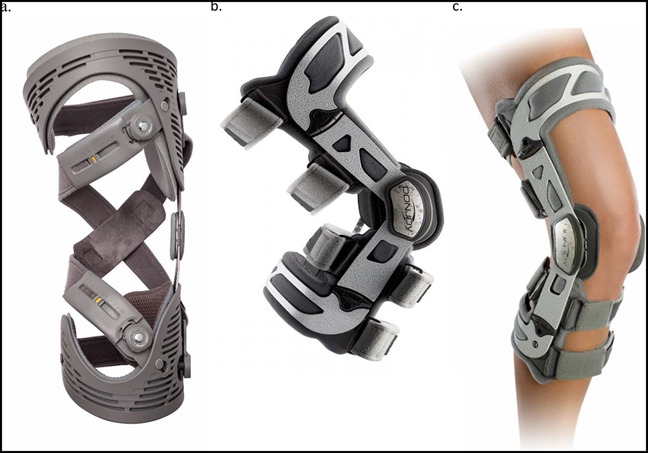
Photographs showing the valgus knee unloader braces (**A**, Ӧssur Unloader One; **B**, DonJoy OA Adjuster 3; and **C**, DonJoy Nano). The brace produces a valgus force with the medial “stay” transferring body weight away from the affected medial compartment to the nonaffected lateral compartment; varus knee unloader brace is used for the affected lateral compartment. The primary indication for unloader braces is knee osteoarthritis. Although all three knee braces treat mild-to-moderate OA, the main differences are weight, fit, and patient comfort which varies between patients (permission to use these photographs were received from Ӧssur, Reykjavík, Iceland, and DJO Lewisville, TX).

The mechanisms of action for unloader knee braces vary depending on the location of osteoarthritis that the brace is treating. The brace is designed to adjust alignment appropriately to unload the articular compartment that is causing pain and dysfunction. As such, reported symptoms should be ideally localized to that compartment. The medial unloader knee brace applies a valgus moment for medial compartment osteoarthritis, whereas the lateral unloader knee brace applies a varus moment for lateral component osteoarthritis.^20^ Examples of unloader knee braces are the Össur Unloader One OA (Össur), DonJoy OA Adjuster 3 (DJO), and DonJoy Nano (DJO), which are recommended for mild-to-severe unicompartmental osteoarthritis (Figure 8).

**Figure 9:**
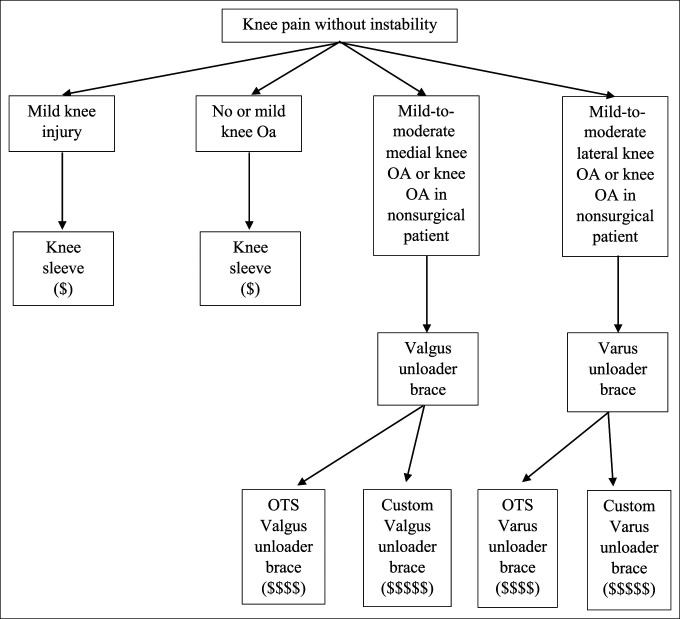
Flowchart showing the knee pain without instability treatment schematic.

A prospective cohort study by Lee et al.^21^ of 63 patients with unicompartmental osteoarthritis demonstrated that unloader knee braces improved the quality of life for the patients. In addition, a prospective cohort study by Lamberg et al.^22^ of 15 patients showed that unloader bracing treatment can reduce knee-knee and second peak knee adduction moments. Unloader braces have the potential to delay or potentially avoid the need for TKA. One randomized control trial included 150 patients who were randomly assigned to a control and placebo group or an unloader group demonstrated the effectiveness of the brace in unloading the affected compartment.^23^ However, given the lack of more high-quality prospective data, limited evidence exists on the effectiveness of unloader braces in managing knee osteoarthritis.^24^ Figure 9 illustrates a treatment schematic for unloader braces.

### Patellofemoral

Patellofemoral knee braces decrease knee pain and resist lateral displacement of the patella for patellofemoral joint-related pain. Repetitive overuse or microtrauma injuries, abnormal patellar tracking, and articular cartilage injury caused by direct trauma can lead to the development of patellofemoral pain. The brace aims to correct and maintain patellar tracking by resisting its lateral displacement. These braces may also be known as a “J-brace” if support is only lateral based or a “donut” brace if the support is circumferential based.

**Figure 10:**
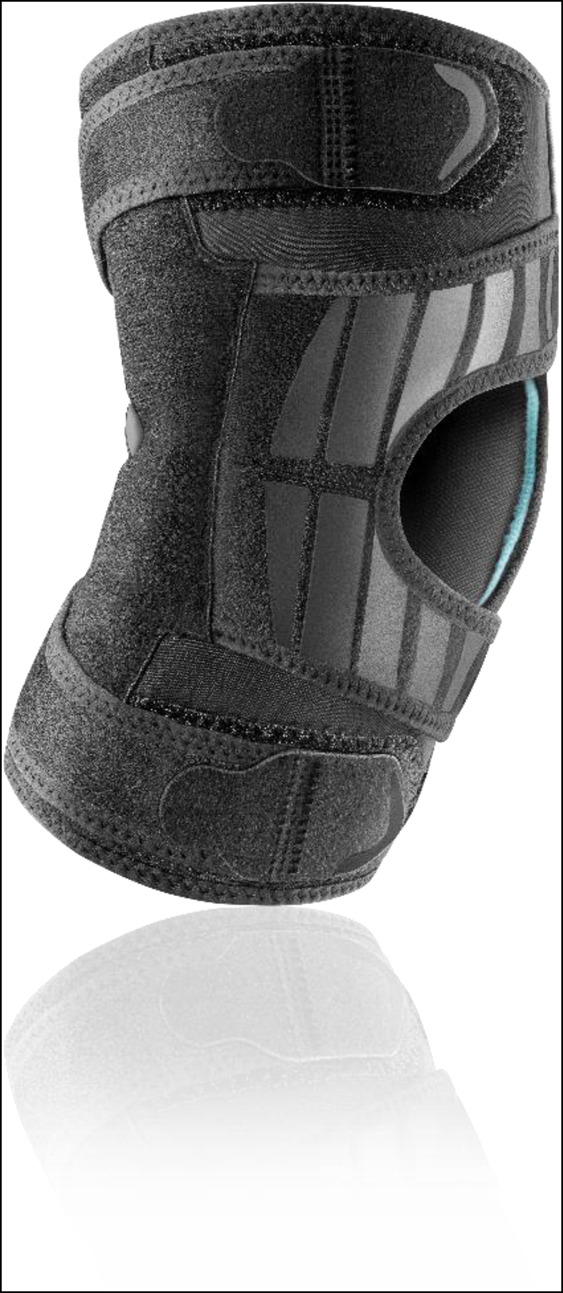
Photograph showing the patellofemoral brace (Ӧssur Formfit Pro Knee Tracker). Most braces have a relief or cutout for the patella with a lateral checkering (hook and loop fastener, eg, Velcro) to prevent additional lateral subluxation of the patella. The primary indication for patellofemoral braces is patellofemoral pain (permission to use this photograph was received from Ӧssur, Reykjavík, Iceland).

The mechanisms of action for patellofemoral knee braces lack consensus. However, the theories for how the brace reduces knee pain include adjusting patellar tracking or alignment by improving the Q angle. This, in turn, may lead to increased patellofemoral contact area to distribute forces more evenly and decrease point loading in an area of osteoarthritis, unloading the extensor mechanism of the knee, altering patellofemoral positioning, and better distribution of forces on the lateral aspects of the patella.^25,26,27,28,29,30^ They have also been postulated to improve circulation, temperature, or proprioception^31^ The braces are often made of flexible material and consist of straps or more robust material in the form of a “J” or “donut” for patellar stabilization. One example of patellofemoral braces is Formfit Pro Knee Tracker (Össur), shown in Figure 10.

**Figure 11:**
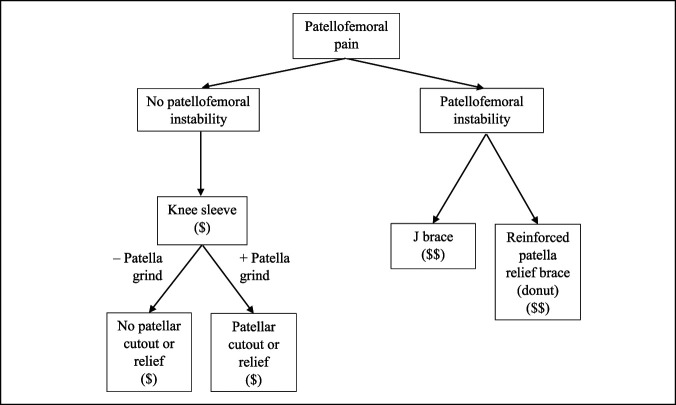
Flowchart showing the patellofemoral brace treatment schematic.

Because of the lack of agreement on the exact mechanism of action for patellofemoral knee braces, their efficacy is unclear. A randomized control trial by Timm KE of 100 patients who were divided int a control group and patellofemoral pain treatment group found that bracing treatment improves patellofemoral position, function, and pain.^32^ However, a prospective cohort study by Miller et al^33^ of 51 patients with anterior knee pain reported no differences between patients with and without patellofemoral knee braces. Figure 11 illustrates a treatment schematic for patellofemoral braces.

### Rehabilitative

According to the AAOS, rehabilitative knee braces allow controlled and secure motion of injured or surgically repaired knees. The braces provide either immobilization of the knees or controlled flexion and extension in predetermined arcs to control excessive varus-valgus rotation and range of motion.^34^

The mechanism of action for rehabilitative knee braces commonly relies on a hinge design, which includes straps, stays, shells, and hinges that interact to function as one unit. More effective rehabilitative knee braces interface the hinge bar and the shells to control limb motion. The presence of shells, material properties of the shell, alignment of the supportive hinges, and location and number of straps influence their mechanical properties.^34^

Rehabilitative braces can be classified into three mechanisms: dynamic bracing treatment, turnbuckle, and static progressive stretch. Dynamic braces, which often function unidirectionally, apply a constant, low-intensity force on the knee joint postoperatively. Commonly, one device is needed for extension, and another device is needed for flexion. Turnbuckle braces, whose tension levels can be altered as needed, provide low-intensity force on the knee joint for contracture release. Static progressive stress braces, which often function bidirectionally, apply a low-intensity force that is increased over time as tissues in the knee joint relax.^35^ The most basic example of progressive braces is the knee immobilizer shown in Figure 3, but progressive braces can also include the functional braces, one of which is shown in Figure 4.

**Figure 12:**
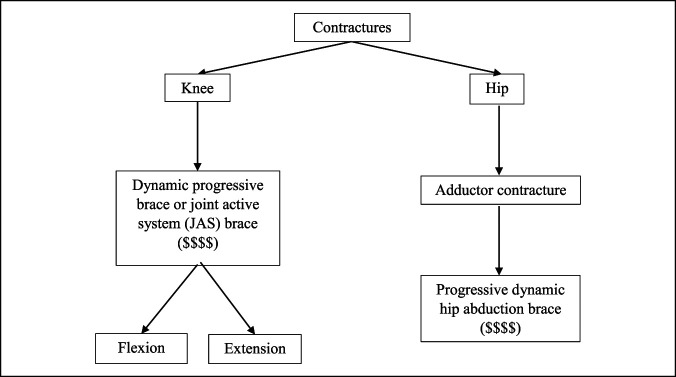
Flowchart showing the hip and knee braces for the contractures treatment schematic.

Studies have shown that static progressive stretch bracing treatment is able to markedly improve range of motion, stiffness, swelling, and pain.^35^ However, there is a lack evidence that demonstrates consistent efficacy of rehabilitative knee braces. A prospective cohort study by Möller et al.^36^ of 62 patients who were randomly divided into a group given postoperative braces or into a group that was not given postoperative braces showed that there is no benefit of using a rehabilitative knee brace postoperatively at any time point up to 24 months. For nonsurgical treatment, other studies have found that despite knee immobilization, patients have persistent instability of their injured knee.^37^ Currently, there is a lack of consensus regarding the frequency, intensity, type, and length of postoperative therapy protocols.^38^ Figure 12 illustrates a treatment schematic for rehabilitative braces.

### Sleeve

Knee sleeves are very commonly used by patients with and without medical input. Knee sleeves can be used in acute injuries as part of rest, ice, and compression protocol or during symptomatic exacerbation of preexisting osteoarthritis. In addition, the knee sleeves can help reduce pain and dysfunction day-to-day or during sporting activities.

**Figure 13:**
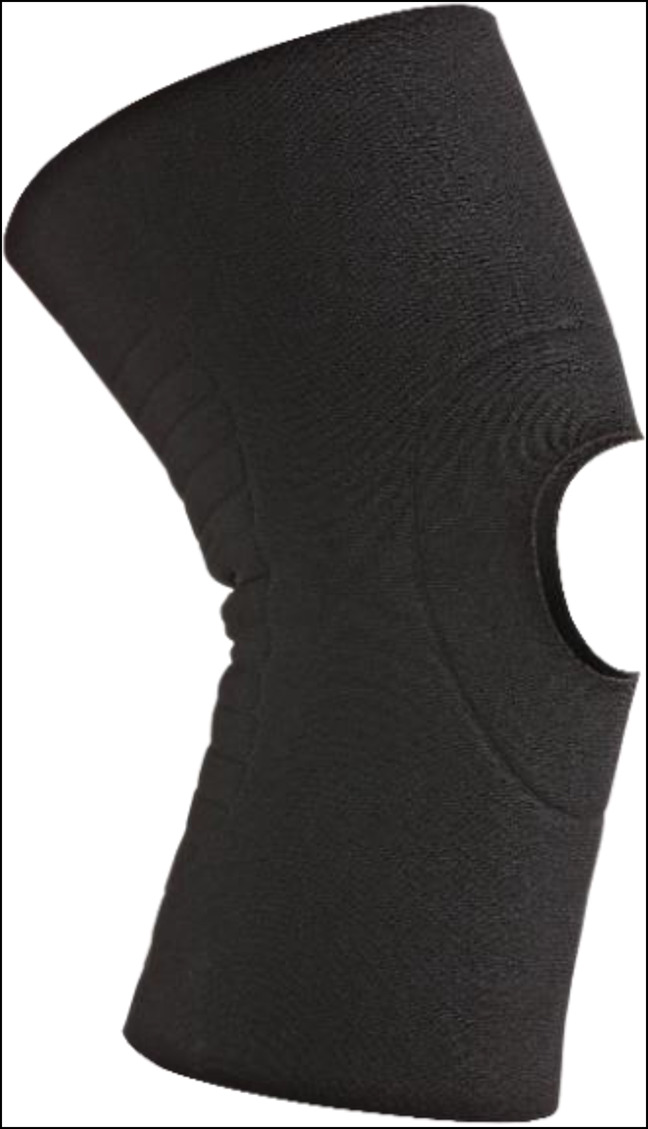
Photograph showing the neoprene knee sleeve (Ӧssur Neoprene Knee Sleeve 1). These are usually the cheapest knee braces and can be easily purchased by patients. Neoprene knee sleeves come with a variety of options for the patella, including with or without patella relief (permission to use this photograph was received from Ӧssur, Reykjavík, Iceland).

Knee sleeves do not provide any structural support; however, they do offer compression to potentially help prevent and reduce a joint effusion.^39^ In addition to general low-level symptomatic relief, they may also provide proprioceptive feedback. One example of a knee sleeve is the Neoprene Knee Sleeve 1 (Össur), shown in Figure 13.

Studies on athlete populations have shown that knee sleeves positively affect neuromuscular control by reducing knee flexion, abduction movement, and adduction moment.^39,40^ Other studies, such as that by Moon et al^40^ of 19 patients, used a musculoskeletal modeling analysis to demonstrate that sleeves do not reduce force transmission through the ACL, knee joint shear force, or internal rotation.

### Orthotist

Although most hip and knee braces are OTS, custom braces will require the use of a brace fitter or orthotist. An orthotist can also assist surgeons on brace choice according to the diagnosis and treatment goals, cost, and patient factors which include activity level, expectations, body habitus, and insurance coverage. For example, the functional brace for a patient with an MCL reconstruction after TKA has different requirements than does the athlete status post-MCL reconstruction. Furthermore, it may not be possible to use certain brace types in patients with larger and/or disproportionately sized limbs, or if there is a notable out of pocket expense required.

In addition to recommending brace types, an orthotist can also assist with custom use and construction of specialty braces. For example, one of our senior authors has had success with a custom “drop lock” knee brace. The drop lock brace was manufactured by an orthotist for patients who could not undergo surgery for extensor mechanism injuries often in the presence of TKA. The orthotist incorporated a lever in the brace that the patient could release in their pocket to unlock the knee when sitting and then lock in full extension for ambulation.

## Summary

Although routinely used, knee and hip brace use has only low-level evidence. However, this absence of clinical evidence should not undermine their clinical importance because they are a relatively simple, widely available, well-tolerated, and noninvasive treatment option for patients. We therefore recommend clinicians to keep our bracing treatment algorithms and figures in their clinics to help make a quick and decisive decision on which brace to use. There are many different brace manufactures and designs, so the involvement of an orthotist is useful to provide the appropriate brace option including more custom designs. Although double-blinded randomized controlled trials of braces are not feasible, more simple studies of patient-reported outcomes with the use of unloader braces or rehabilitation metrics with bracing treatment after ligament surgery are needed.
